# Recurrent Respiratory Syncytial Virus Infection in a CD14-Deficient Patient

**DOI:** 10.1093/infdis/jiac114

**Published:** 2022-04-16

**Authors:** Sjanna B Besteman, Emily Phung, Henriette H M Raeven, Gimano D Amatngalim, Matevž Rumpret, Juliet Crabtree, Rutger M Schepp, Lisa W Rodenburg, Susanna G Siemonsma, Nile Verleur, Rianne van Slooten, Karen Duran, Gijs W van Haaften, Jeffrey M Beekman, Lauren A Chang, Linde Meyaard, Tjomme van der Bruggen, Guy A M Berbers, Nicole Derksen, Stefan Nierkens, Kaitlyn M Morabito, Tracy J Ruckwardt, Evelyn A Kurt-Jones, Douglas Golenbock, Barney S Graham, Louis J Bont

**Affiliations:** Center for Translational Immunology, University Medical Centre Utrecht, Utrecht, the Netherlands; Department of Pediatrics, Wilhelmina Children’s Hospital, University Medical Centre Utrecht, Utrecht, the Netherlands; Vaccine Research Center, National Institute of Allergy and Infectious Diseases, National Institutes of Health, Bethesda, Maryland, USA; Department of Pediatric Pulmonology, Wilhelmina Children’s Hospital, University Medical Center Utrecht, Utrecht University, Utrecht, the Netherlands; Regenerative Medicine Center Utrecht, University Medical Center Utrecht, Utrecht University, Utrecht, the Netherlands; Department of Pediatric Pulmonology, Wilhelmina Children’s Hospital, University Medical Center Utrecht, Utrecht University, Utrecht, the Netherlands; Regenerative Medicine Center Utrecht, University Medical Center Utrecht, Utrecht University, Utrecht, the Netherlands; Center for Translational Immunology, University Medical Centre Utrecht, Utrecht, the Netherlands; Oncode Institute, Utrecht, the Netherlands; Department of Infectious Diseases and Immunology, Department of Medicine, University of Massachusetts Medical School, Worcester, Massachusetts, USA; National Institute of Public Health and the Environment (RIVM), Bilthoven, the Netherlands; Department of Pediatric Pulmonology, Wilhelmina Children’s Hospital, University Medical Center Utrecht, Utrecht University, Utrecht, the Netherlands; Regenerative Medicine Center Utrecht, University Medical Center Utrecht, Utrecht University, Utrecht, the Netherlands; Center for Translational Immunology, University Medical Centre Utrecht, Utrecht, the Netherlands; Department of Pediatrics, Wilhelmina Children’s Hospital, University Medical Centre Utrecht, Utrecht, the Netherlands; Center for Translational Immunology, University Medical Centre Utrecht, Utrecht, the Netherlands; Department of Genetics, Center for Molecular Medicine, University Medical Center Utrecht, Utrecht, the Netherlands; Department of Genetics, Center for Molecular Medicine, University Medical Center Utrecht, Utrecht, the Netherlands; Department of Pediatric Pulmonology, Wilhelmina Children’s Hospital, University Medical Center Utrecht, Utrecht University, Utrecht, the Netherlands; Regenerative Medicine Center Utrecht, University Medical Center Utrecht, Utrecht University, Utrecht, the Netherlands; Vaccine Research Center, National Institute of Allergy and Infectious Diseases, National Institutes of Health, Bethesda, Maryland, USA; Center for Translational Immunology, University Medical Centre Utrecht, Utrecht, the Netherlands; Oncode Institute, Utrecht, the Netherlands; Department of Medical Microbiology, University Medical Center Utrecht, Utrecht, the Netherlands; National Institute of Public Health and the Environment (RIVM), Bilthoven, the Netherlands; RSV Patient Network, ReSVinet, Zeist, the Netherlands; Center for Translational Immunology, University Medical Centre Utrecht, Utrecht, the Netherlands; Vaccine Research Center, National Institute of Allergy and Infectious Diseases, National Institutes of Health, Bethesda, Maryland, USA; Vaccine Research Center, National Institute of Allergy and Infectious Diseases, National Institutes of Health, Bethesda, Maryland, USA; Department of Infectious Diseases and Immunology, Department of Medicine, University of Massachusetts Medical School, Worcester, Massachusetts, USA; Department of Infectious Diseases and Immunology, Department of Medicine, University of Massachusetts Medical School, Worcester, Massachusetts, USA; Vaccine Research Center, National Institute of Allergy and Infectious Diseases, National Institutes of Health, Bethesda, Maryland, USA; Center for Translational Immunology, University Medical Centre Utrecht, Utrecht, the Netherlands; Department of Pediatrics, Wilhelmina Children’s Hospital, University Medical Centre Utrecht, Utrecht, the Netherlands

**Keywords:** CD14, epithelium, monocyte, respiratory syncytial virus, Toll-like receptor

## Abstract

**Background:**

Recurrent respiratory syncytial virus (RSV) infection requiring hospitalization is rare and the underlying mechanism is unknown. We aimed to determine the role of CD14-mediated immunity in the pathogenesis of recurrent RSV infection.

**Methods:**

We performed genotyping and longitudinal immunophenotyping of the first patient with a genetic CD14 deficiency who developed recurrent RSV infection. We analyzed gene expression profiles and interleukin (IL)-6 production by patient peripheral blood mononuclear cells in response to RSV pre- and post-fusion (F) protein. We generated CD14-deficient human nasal epithelial cells cultured at air-liquid interface (HNEC-ALI) of patient-derived cells and after CRISPR-based gene editing of control cells. We analyzed viral replication upon RSV infection.

**Results:**

Sanger sequencing revealed a homozygous single-nucleotide deletion in *CD14*, resulting in absence of the CD14 protein in the index patient. In vitro, viral replication was similar in wild-type and *CD14*^*−*/−^ HNEC-ALI. Loss of immune cell CD14 led to impaired cytokine and chemokine responses to RSV pre- and post-F protein, characterized by absence of IL-6 production.

**Conclusions:**

We report an association of recurrent RSV bronchiolitis with a loss of CD14 function in immune cells. Lack of CD14 function led to defective immune responses to RSV pre- and post-F protein without a change in viral replication.

## Teaser

Autosomal recessive CD14 deficiency is a novel genetic etiology associated with recurrent RSV bronchiolitis. Lack of CD14 function led to defective immune responses to RSV pre- and post-F protein.

Respiratory syncytial virus (RSV) is one of the most important viral pathogens identified in respiratory tract infections (RTIs) in children. It causes a major global health burden, with a hospitalization rate of ~3.2 million and an estimated yearly mortality rate of 125 000 in children under 5 [1]. Most infants who develop severe infection and require admission to the intensive care unit are below the age of 3 months [2]. Recurrent RSV hospitalization is rare and without a known genetic etiology. The recent discovery and stabilization of the pre-fusion (F) conformation of the RSV surface F glycoprotein accelerated vaccine development [3]. Still, designing an effective RSV vaccine or therapy remains challenging [4]. Defining immunological determinants of RSV susceptibility and disease severity may aid the advancement of new therapeutics and vaccine development [4, 5].

Respiratory syncytial virus F is required for infection of respiratory epithelial cells. After entering the respiratory tract, RSV is confronted with the innate antiviral responses mediated by the airway epithelium and lung resident immune cells such as alveolar macrophages [5]. These first molecular events upon infection define the size and direction of the inflammatory response. A dysregulated inflammatory response can lead to increased viral burden, as well as enhanced pulmonary inflammation [5]. Hence, robust innate defenses that partially control virus replication will reduce the level of adaptive immune responses needed to clear infection, reducing immunopathology [6]. Toll-like receptors (TLRs) such as TLR2 and TLR4, and the coreceptor CD14, have been shown to modulate the immune response to RSV and accelerate viral clearance [7–9]. The CD14 receptor exists in 2 forms: membrane bound by a glycosylphosphatidylinositol anchor to immune and epithelial cells (mCD14) and soluble (sCD14), circulating in the blood and at mucosal sites [10]. CD14 functions primarily as a lipopolysaccharide (LPS) transferase, greatly accelerating the binding of LPS to a complex consisting of TLR4 and myeloid differentiation factor 2 (MD2) [11, 12]. The association of CD14 function with RSV disease outcome remains controversial, and the clinical relevance of CD14 as part of the putative RSV receptor complex has not been shown [13–16]. It has even been proposed that RSV evolved the ability to stimulate the CD14-TLR4-MD2 axis for its own benefit [13]. Over the years, the role of CD14 has been extensively studied in cell lines and animal models, but a human model has yet to be described. We describe the first child with a homozygous frameshift mutation in *CD14*, leading to autosomal recessive CD14 deficiency, as a novel genetic etiology associated with recurrent RSV bronchiolitis. The accidental identification of a CD14-deficient patient enabled us to investigate the role of the CD14-mediated immune response in the context of RSV infection. We aimed to confirm the role of human CD14 expressed by monocytes in response to RSV-F stimulation. Next, we studied whether RSV viral replication in vitro is affected by the absence of CD14 in airway epithelial cells.

## MATERIAL AND METHODS

### Study Participants

The patient and infant controls were initially enrolled through a study previously performed by our group (Neon Study: Institutional Review Board review reference number NL58404.041.16). In short, the Neon study was set up to study the phenotype and function of airway and blood-derived neutrophils of patients with severe bronchiolitis [17] All participants gave written informed consent. The Parent Advisory Board of our RSV research team was involved in writing a patient information letter specifically for the index patient.

### Immunophenotyping

We performed flow cytometry to identify CD14 surface expression on patient and control immune cells. Whole blood was depleted from red blood cells. Cells were stained for surface markers for 20 minutes at 4°C in phosphate-buffered saline (PBS) containing 0.01% (m/v) sodium azide and 1% (m/v) bovine serum albumin. Cell types were identified according to their characteristic forward and side light-scatter properties and by their typical cell surface markers: neutrophils, CD14^−^/CD16^+^; monocytes, CD14^+^/CD16^−^. 7-aminoactinomycin D was used to distinguish apoptotic cells. Generation of RSV pre-F and post-F probes and identification of RSV pre-F and post-F binding B cells were performed as described previously [3].

### Monocyte Isolation and Stimulation

Peripheral blood mononuclear cells (PBMCs) were extracted from whole blood using Ficoll. Monocytes were isolated from the PBMCs by Percoll gradient. Standard Isotone Percoll (SIP) was prepared by adding 10:1 10× PBS. The PBMCs were taken up in 60% SIP, followed by gentle addition of 47.5% and 34% SIP, centrifuged for 45 minutes, 1750 relative centrifugal force, at room temperature. Monocytes were collected from the upper ring and washed twice with PBS. After isolation, PBMCs or monocytes were stimulated with a panel of Toll-like receptor (TLR) agonists. All TLR stimuli were obtained from InvivoGen, unless stated otherwise. Where indicated, monocytes were preincubated for 30 minutes at 4°C with 10 µg/mL anti-hCD14-IgG (InvivoGen) or mouse antihuman HLA-ABC (BD Pharmingen) as control. Polymyxin B 10 µg/mL (InvivoGen) was added to the PBMCs 10 minutes before the addition of LPS or RSV F. For heat inactivation, LPS and RSV F were heated for 10 minutes at 99°C before adding this to the PBMCs. All assays with cells derived from the index patient were performed within the age range of 10 months to 3 years.

### Collection and Differentiation of Nasal Epithelial Cells

Primary human nasal epithelial cells (HNECs) were obtained from (1) healthy children and the (2) index patient by nasal brushes. All participants or their caregivers provided written informed consent. The study was approved by a specific ethical board for the use of biobanked materials TcBIO (Toetsingscommissie Biobanks), an institutional Medical Research Ethics Committee of the University Medical Center Utrecht (protocol ID: 19/678). Nasal airway cells were collected, isolated, expanded and differentiated as previously described [18]. CD14 knockout cell lines were made with CRISPR-Cas9 technologies as described previously [19–21].

### Viral Infection of Human Nasal Epithelial Cells Cultured at Air-Liquid Interface

One day before infection, culture medium of the HNEC cultured at air-liquid interface (HNEC-ALI) was refreshed. RSV-A2-GFP was diluted in serum-free Opti-MEM (Gibco) to obtain a multiplicity of infection (MOI) of 1. The HNEC-ALI were infected at the apical side with 100 μL RSV-A2-GFP or serum-free Opti-MEM only (mock).

### Respiratory Syncytial Virus Immunoglobulin G Concentrations and Avidity Assays

Analysis of immunoglobulin (Ig)G in serum was performed using an RSV multiplex immunoassay as described earlier. The avidity of the RSV-specific antibodies was determined using the same RSV multiplex immunoassay with some adaptations. After incubation of the sera with the RSV proteins, all samples were additionally incubated for 10 minutes, at room temperature, in 3-fold, on the same plate, in the presence of 1.5 M ammonium thiocyanate (NH_4_SCN), 3.0 M NH_4_SCN, or PBS (pH 7.4). The avidity index was expressed as the percentage of residual mean fluorescence intensity IgG signal in comparison to the undenatured (PBS) signal, which was set at 100%.

### Respiratory Syncytial Virus Neutralization Assays

Neutralization by sera from the index patient, 15 adult controls of a previous study [22], and the BEI NR-4020 control sera were measured by a fluorescence plate reader neutralization assay described previously [3].

### Statistical Analysis

Statistical analysis was performed with GraphPad Prism software version 8.3.0. An unpaired *t* test was performed to compare patient groups or samples. *P* < 0.05 were considered significant. Additional information on the applied methodologies can be found in this article’s online Supplement.

## RESULTS

### Clinical Phenotype of CD14 Deficiency

**Figure 1.**
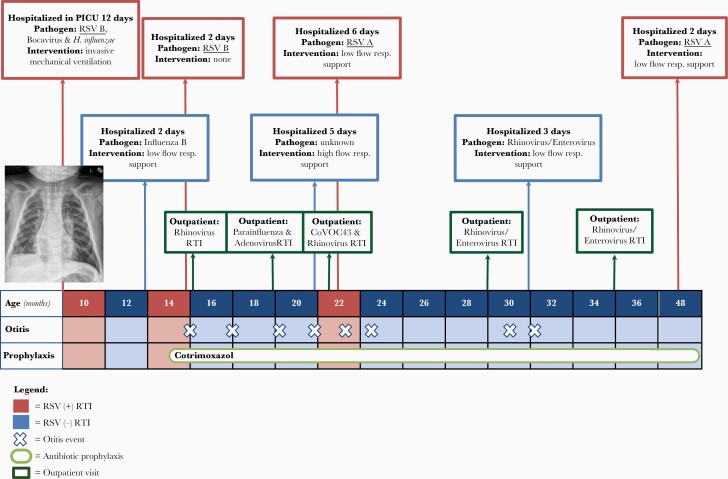
Overview of the patient’s medical history. Timeline of critical events in the patient’s medical history, according to his age in months. CoVOC43, human coronavirus OC43; CRP, C-reactive protein; RSV, respiratory syncytial virus; RTI, respiratory tract infection; resp, respiratory.

**Figure 2.**
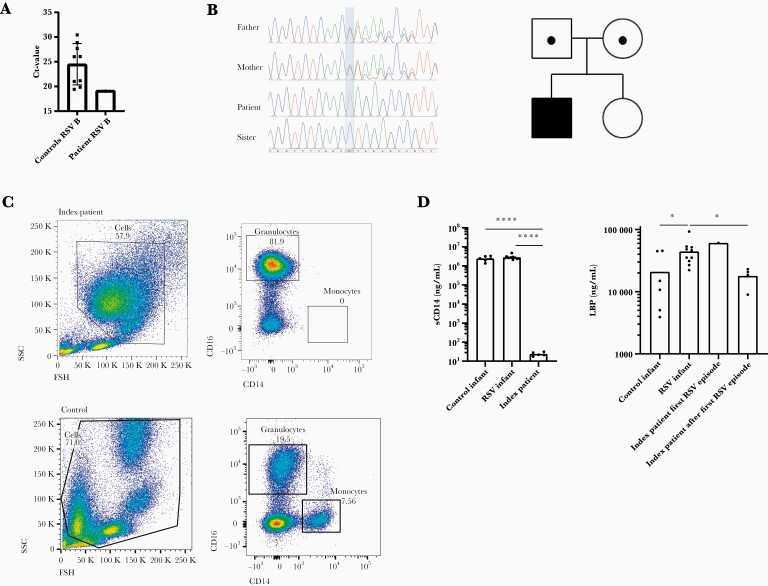
Phenotypic and genetic analysis of CD14 deficiency. (a) Respiratory syncytial virus (RSV) cycle threshold (Ct) value of the index patient during the first RSV infection and of 9 controls admitted with RSV at the pediatric intensive care unit in the same season. Mean + standard deviation is shown for the controls. (b) Genomic deoxyribonucleic acid sequence of *CD14* comparing the patient (*CD14*^−/−^) with his parents (both *CD14*^*+/−*^) and sister (wild type). The band indicates the deletion of C in the index patient. In the family pedigree, circles and squares denote female and male family members, black dots represent a heterozygous mutation in *CD14*, and the black square represents the index patient. (c) Flow cytometry plot of the index patient. Cells were gated based on their forward and side scatter (SSC) properties, and single, nonapoptotic cells were selected. Monocytes were identified a CD14^+^/CD16^−^. (d) Plasma levels of soluble CD14 (sCD14) in the index patient compared to control infants (n = 6) and RSV infants (n = 10). Levels of lipopolysaccharide-binding protein (LBP) during and after the first RSV episode in the index patient, compared to control infants (n = 6) and RSV infants (n = 10). An unpaired *t* test was performed to compare expression between patient groups. Only significant values are shown. *, *P* = <.05; ****, *P* = <.0001. (^−/−^), genetic knock out.

The index patient, a boy, had life-threatening RSV type B bronchiolitis at the age of 9 months (Figure 1). He required intubation and invasive mechanical ventilation at the pediatric intensive care unit (PICU) for a period of 11 days. His RSV cycle threshold (Ct) value was 19.18, and control infants admitted to the PICU with severe RSV had a mean RSV Ct value of 24.51 (*+*standard deviation [SD] 4.2) (Figure 2a), measured in broncho tracheal aspirates. He had 3 additional episodes of RSV bronchiolitis at the age of 14 and 22 months and 4 years, which required hospital admission for observation of the respiratory status and supplemental oxygen supply. The patient tested negative for RSV between episodes. At the time of the first RSV episode, we discovered the patient’s CD14 deficiency by studying the patient’s leukocytes via flow cytometry (Supplementary Figure S1) [23]. This was confirmed by Sanger sequencing, which showed a single-nucleotide deletion in the *CD14* gene: a homozygous variant (NM_000591.4:c.196del), resulting in a frameshift and truncation in the CD14 protein (NP_000582.1:p.Leu66*) (Figure 2b and Supplementary Figure S2). This rare variant is present in the European (non-Finnish) population with an allele frequency of 4.54 × 10^−^5 and is absent in the homozygous state (gnomAD). Both parents were heterozygous for the mutation, and the sister had a normal genotype. Both parents had normal CD14 cell surface expression (Supplementary Figure S3). No other de novo or recessive variants were identified that score as variant of uncertain significance or higher according to the 2015 American College of Medical Genetics and Genomics-Association for Molecular Pathology guidelines [24]. The child was admitted 3 times with non-RSV respiratory tract infections, including human rhinovirus (HRV)/enterovirus infection. Figure 1 shows an overview of the events in the patient’s medical history, and an extended case report is presented in an online repository. Altogether, the clinical phenotype of CD14 deficiency is characterized by respiratory infections, in particular recurrent RSV bronchiolitis, requiring hospital admission.

### Immunological Phenotype of Human CD14 Deficiency

Evaluation of patient’s leukocytes by flow cytometry showed no CD14 cell surface expression (truncated or intact), and there was no intact sCD14 detected in the plasma (Figure 2c and d). In case a truncated form of CD14 is present in the circulation, we predicted this would not be functional because it lacks the pocket rim needed for LPS binding (Supplementary Figure S2b) [25]. We measured LPS-binding protein (LBP) levels in patient plasma during his first RSV episode and at several time points thereafter. Our index patient, and other infants hospitalized with severe RSV, had mildly increased LBP levels compared to control infants. After the first RSV episode, the index patient had normal LBP levels (Figure 2d). To investigate the local inflammatory response during RSV infection in vivo, we measured levels of IL-6 and IL-8 in sputum of the index patient and controls. The levels of IL-6 and IL-8 in sputum of the index patient fell within the range of the control patients (Supplementary Figure S4). In addition, because neutrophils are the main immune cell in the airways during RSV infection, we analyzed neutrophil responses, as investigated in a previous study by our group [23]. The mean percentage of airway neutrophils was slightly higher in the index patient (77.1%) compared to control infants with RSV (56.9%; *+*SD 24). The patient and controls showed a similar increase of CD66b and decrease of CD62L surface expression on RSV airway neutrophils compared with circulating neutrophils during severe RSV infection. In addition, oxidative burst by the patient neutrophils was similar to that of control infants (data not shown). In conclusion, local neutrophil function during RSV infection was similar in the index patient and control infants.

### CD14 Deficiency Leads to Impaired Toll-Like Receptor Signaling

**Figure 3.**
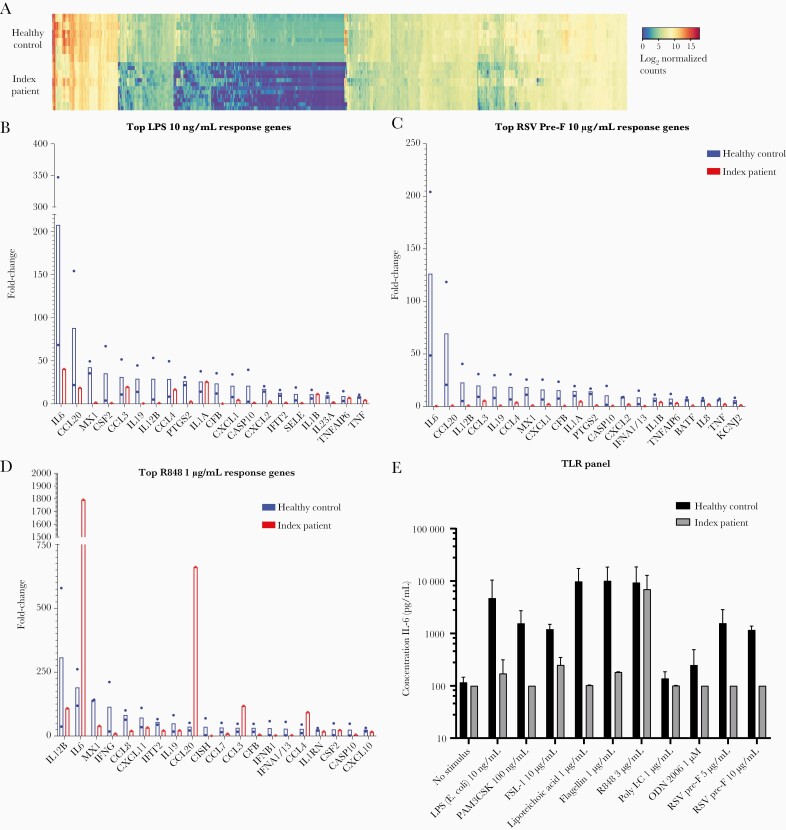
Impaired innate immune response to Toll-like receptor (TLR) 1/2, 2/6, 4, and 5 stimulation in CD14 deficiency. (a) Gene expression by peripheral blood mononuclear cells (PBMCs) after 4 hours of stimulation with a panel of TLR ligands, quantified using NanoString Technology. Log_2_ normalized counts are represented for the index patient (top) and a healthy control (bottom). Upregulated genes are depicted in gray, and downregulated genes are depicted in black. Each row represents a stimulus. Each column represents 1 gene. In Supplemental Figure S9, a large version of the heatmap can be found. (b–d) Expression of the top 20 response genes measured in a, after stimulation with lipopolysaccharide (LPS) (*Escherichia coli* [E. coli]) (10 ng/mL), R848 (1 µg/mL), and respiratory syncytial virus (RSV)-pre-fusion (pre-F) (10 µg/mL). Fold change compared with unstimulated is shown for a healthy control ([HC] black bars) and the index patient (gray bars). (e) Interleukin (IL)-6 protein expression by patient and control PBMCs (n = 4 donors) after TLR stimulation. Values show the mean + standard deviation of 2 independent experiments.

Next, we studied (1) the functionality of CD14.-dependent signaling pathways by stimulating PBMCs of the patient and (2) healthy adult controls with TLR agonists, and we measured innate immune gene expression by NanoString, a methodology for measuring messenger ribonucleic acid (mRNA) levels in the absence of amplification. The innate immune response to TLR 1/2, 2/6, 4, and 5 but not TLR 7/8 stimulation was impaired in the patient’s PBMCs compared to healthy controls (HCs) (Figure 3a–c). This included an absent *IL-6* mRNA response to RSV-pre-F in the index patient (Figure 3d). In addition, mRNA expression of several chemokines (*CCL2*, *CCL20*) and interferon (IFN)-stimulated genes (*MX1*, *IFNa 1/13*) were compromised (Figure 3d). Subsequent protein measurement of IL-6 in supernatant confirmed the *IL-6* mRNA data (Figure 3e). In line with these results, prolonged stimulation of patient-derived PBMCs (Supplementary Figure S5) with LPS, but not the TLR7/8 ligand R848, with varying concentrations, showed impaired IL-6 protein production. We conclude that this patient, with a homozygous autosomal recessive CD14 deficiency, demonstrated a loss of known CD14 functions.

### Airway Epithelial Derived CD14 Does Not Inhibit Respiratory Syncytial Virus Infection

**Figure 4.**
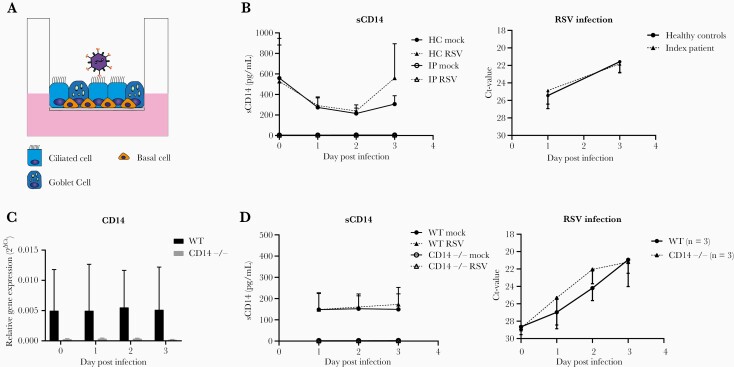
Airway epithelial derived CD14 does not inhibit respiratory syncytial virus (RSV) infection. (a) Schematic view of RSV infection of human nasal epithelial cells cultured at air liquid interface (HNEC-ALI). The HNEC-ALI was derived from healthy control (HC) children (n = 2), the index patient (IP), wild type ([WT] n = 4), and CD14 (n = 4). (b) Cultures were infected with RSV-A2-GFP. Cytokine production of soluble (s)CD14 at day 1, 2, and 3 postinfection in cultures of the index patient and controls is shown. No significant difference in cycle threshold (Ct) value was observed between the index patient and controls (*P* > .05 at both time points). (c) Plots show mRNA expression of CD14. We confirmed the absence of CD14 mRNA expression in CD14 knock out (CD14^−/−^) cultures. (d) Cultures were infected with RSV-A2-GFP. Cytokine production of soluble CD14 (sCD14) at day 1, 2, and 3 postinfection in WT and CD14^−/−^ cultures is shown. No significant differences in cycle threshold (Ct) value were observed between WT and CD14^−/−^ cultures (*P* > .05 for all time points). Values show the mean + standard deviation of 4 donors. The experiment using patient-derived HNEC-ALI were performed twice. The experiments using HNEC-ALI with WT and CD14^−/−^ was performed twice, using 2 individual donors per experiment. All experiments were performed in duplicate. An unpaired *t* test was performed to compare expression (1) between the index patient and controls and (2) between WT and CD14^−/−^. Only significant values are shown.

Next, we studied whether RSV viral replication in vitro is affected by the absence of CD14 in airway epithelial cells (Figure 4a). First, we compared RSV infections in HC and CD14-deficient patient-derived ALI-HNEC (Figure 4a). As expected, soluble CD14 protein levels could be detected in HC ALI-HNEC culture supernatants, but not in the supernatants of the CD14-deficient patient (Figure 4b). However, we did not observe differences in RSV replication between HC and CD14-deficient patient-derived cells in time (Figure 4b). We further confirmed the lack of effect of CD14 deficiency on RSV infection in airway epithelial cells by using CD14^−/−^ cells developed with CRISPR-Cas9-based gene editing (Supplementary Figure S6). Corresponding with CD14-deficient patient-derived cells, CD14^−/−^ cells displayed a lack of CD14 expression compared with wild-type (WT) control cells (Figure 4c and d). Furthermore, RSV replication in CD14^−/−^ ALI-HNEC was comparable with WT controls (Figure 4d). Finally, the production of IL-8 was not different in CD14 knockout and WT HNEC-ALI (Supplementary Figure S7). Based on these findings, we conclude that in the absence of immune cells, epithelial-derived CD14 does not inhibit RSV infection in the airway epithelium.

### CD14 Expression on Immune Cells Mediates the Innate Immune Response Against Respiratory Syncytial Virus Fusion Protein

**Figure 5.**
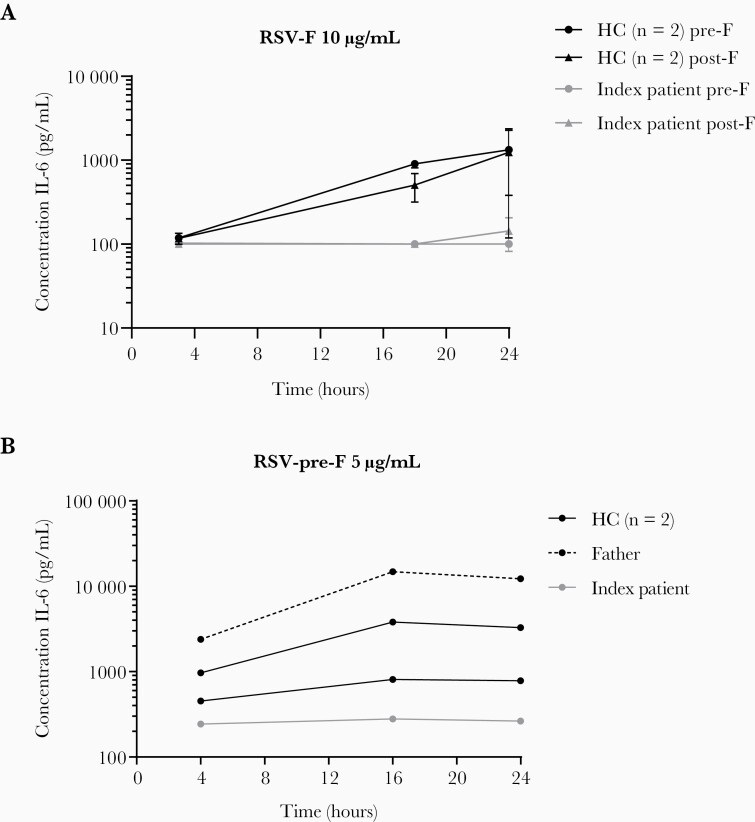
CD14 expression on monocytes mediates the interleukin (IL)-6 response to respiratory syncytial virus fusion (RSV-F). (a) The IL-6 protein response after 4, 18, and 24 hours of stimulation by RSV-pre-F and post-F (10 µg/mL), by peripheral blood mononuclear cells of 2 healthy controls (HC) and the index patient (IP). One experiment, performed in duplicates, lines indicate mean per individual (*+*standard deviation). (b) The IL-6 protein response during 24 hours of stimulation by RSV-pre-F (5 µg/mL) by Percoll-isolated monocytes of 2 healthy controls, the index patient, and his father. One experiment, lines indicate mean per individual.

**Figure 6.**
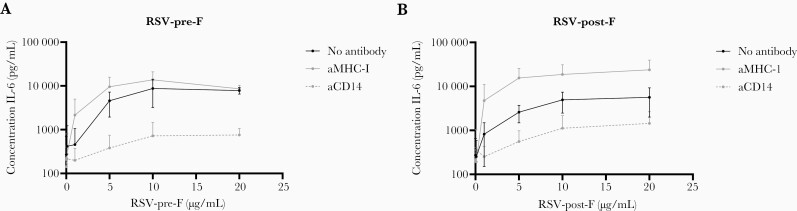
Impaired interleukin (IL)-6 protein response to respiratory syncytial virus fusion (RSV-F) after blocking CD14 in healthy adult donor monocytes. The figure shows IL-6 protein production after stimulation of healthy donor monocytes with RSV pre-F (a) or post-F (b) at different concentrations, by healthy donor monocytes, after Percoll isolation, in the presence of CD14 blocking antibodies (aCD14), control antibody (aMHC-1), or no antibody. The lines indicate the mean (*+*standard deviation) of 3 independent experiments with a total of 7 donors.

**Figure 7.**
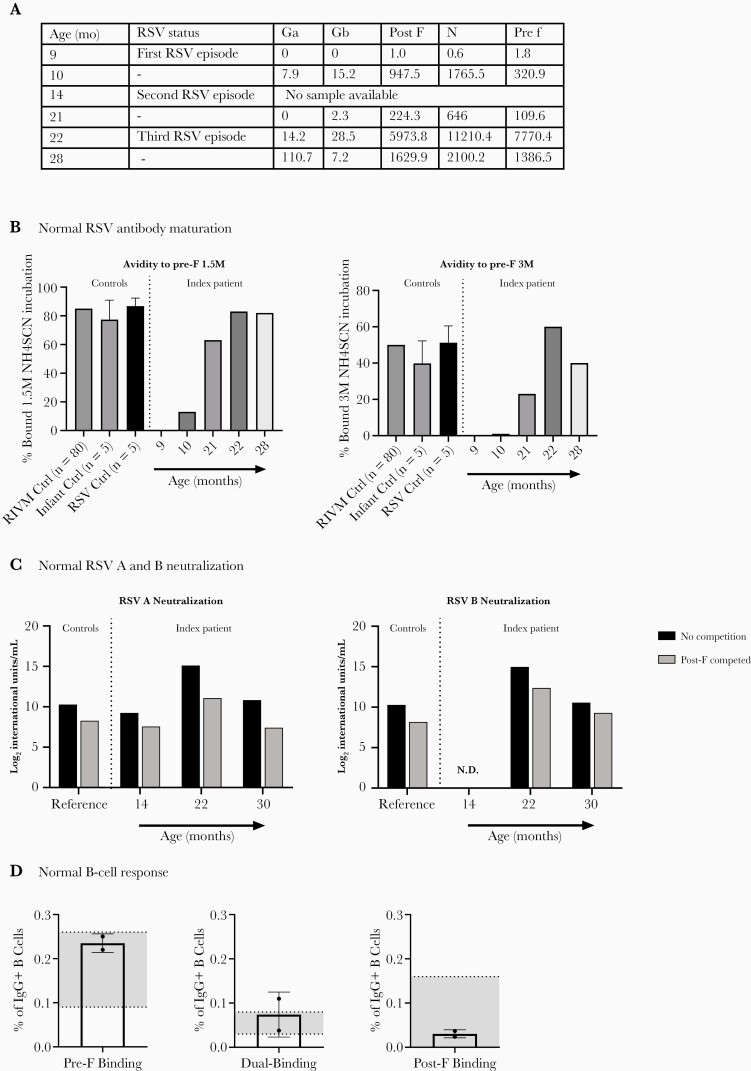
Normal pre-fusion (F)-specific antibody response in CD14 deficiency. (a) Seroconversion of respiratory syncytial virus (RSV)-specific antibodies after the first RSV infection in the index patient. Depicted are the concentrations immunoglobulin (Ig)G against Ga, Gb, post-F, N, and pre-F in arbitrary units/mL at different time points during and in between RSV infections. (b) Percentage pre-F-specific IgG bound after incubation with 1.5M NH4SCN (left graph) and 3M NH4SCN (right graph), at the time points indicated in a. (c) Conformation-specific RSV A and B neutralizing activity in sera obtained at 14, 22, and 30 months, in the absence and presence of excess post-F. Bars represent mean neutralizing activity normalized to International Units of Neutralization. As a reference, the mean neutralization of 15 adult controls is depicted. (d) B-cell specific binding to RSV-pre- and post-F, 8 months after the second RSV infection and 6 months after third RSV infection. Bars show assembled percentage of total IgG + B cells that bind pre-F, post-F, or both probes (dual binding) in the index patient. The interquartile range of healthy controls is depicted in gray. N.D., not determined due to too little available serum; RIVM, Rijksinstituut voor Volksgezondheid en Milieu (National Institute for Public Health and the Environment).

To investigate the role of systemic CD14 in the immune response to RSV F, patient immune cells were stimulated for up to 24 hours with pre-F or post-F protein. This resulted in impaired IL-6 response to both proteins in the patient (Figure 5). In line, surface blockade of CD14 with a monoclonal antibody in healthy donor monocytes inhibited the IL-6 protein response to pre-F and post-F at different concentrations (Figure 6), which confirmed previous observations [8]. To rule out that this response was caused by LPS contamination of RSV F stocks, we measured IL-6 (1) after heat inactivation and (2) in the presence of LPS-neutralizing peptide polymyxin B. After stimulation with heat-inactivated RSV F, IL-6 was not detectable in culture supernatant, whereas it was present after addition of polymyxin B, ruling out LPS contamination (Supplementary Figure S8). In addition, we investigated adaptive immune responses to RSV. The patient had normal seroconversion and concentrations of RSV-specific antibody titers, with normal avidity (Figure 7a–b). Furthermore, the binding and neutralizing activity of total pre-F and pre-F exclusive RSV antibodies in the serum was normal (Figure 7c). Finally, B-cell phenotyping revealed that memory IgG + B cells binding to pre-F, post-F, or both probes fell within the expected range (Figure 7d), demonstrating an intact memory B-cell response [22]. In conclusion, we show that systemic CD14 mediates the innate immune response against RSV infection.

## DISCUSSION

Life-threatening RSV infection at age 9 months, followed by recurrent RSV-related hospitalizations, is rare [2], and a possible genetic predisposition is unknown. Although single-nucleotide polymorphisms (SNPs) in the *CD14* and *TLR4* genes have been associated with severe RSV bronchiolitis, a genetic link to recurrent or severe disease has not been described [26, 27]. We identified the first CD14-deficient patient with an impaired innate immune response against RSV. We show that airway epithelium-derived CD14 did not affect RSV viral infection and that CD14 on immune cells impaired the immune response against RSV. Our data suggest a beneficial role for CD14 in RSV immune signaling, and we extend these findings to a clinical phenotype for CD14 deficiency associated with recurrent respiratory tract infections.

Casanova et al [28] showed that susceptibility to common severe infections can be linked to single-gene inborn errors of innate immunity. Previous TLR-signaling deficiencies linked to disease in humans include the following: TLR3 deficiency as a cause of herpes simplex encephalitis, MyD88-IRAK4 deficiency in pyogenic infections, MDA5 deficiency underlying severe HRV infection, inherited IRF7 and IRF9 deficiencies associated with severe influenza pneumonitis, and TIRAP deficiency as a cause of staphylococcal disease [29–33]. We demonstrated that a deficiency of CD14 was characterized by, but not limited to, recurrent RSV infections. The patient was also hospitalized with RTIs caused by other viruses, including rhinovirus. Although recurrent rhinovirus infection is common in childhood [34], it could be that recurrent viral RTI’s are part of the CD14 deficiency phenotype. Thus far, a link between CD14 and these viruses has not been reported in literature. In vitro, the patient’s immune cells showed an impaired innate immune response to the RSV F protein. Lung resident cells, including airway epithelial cells, alveolar macrophages, and dendritic cells, together with infiltrating monocytes and neutrophils initiate the innate immune response against RSV [5, 35]. Impaired innate immune responses by monocytes, as in our patient, are likely to affect the host’s susceptibility to RSV. This is supported by our previous observation that SNPs in innate immune genes are related to predisposition to RSV infection [36]. Our data show that without CD14-mediated engagement of innate immune responses, adaptive immunity to RSV may be inadequate to control viral replication and protect from recurrent RSV disease.

The mechanism by which CD14 affects IL-6 signaling in response to RSV-F and how this is linked to the clinical phenotype remains to be elucidated. First, we showed impaired monocyte immune responses, characterized by impaired IL-6 production in vitro. Second, IL-6 and IL-8 responses during severe RSV infection in sputum in the CD14-deficient patient were similar to control infants. Third, we show normal neutrophil function and influx to the airway. Fourth, there are no clinical or biochemical signs of chronic inflammation. Based on our data, we hypothesize that an impaired CD14-mediated IL-6 response results in decreased viral protection, and that CD14 plays a limited role in neutrophil induced immune pathology or chronic inflammation.

In humans, CD14 has been shown to interact with TLR2 and TLR4 [37], whereas in mice CD14 also associates with TLR3, TLR7, and TLR9 [38, 39]. We identified an impaired immune response to agonists against TLR1/2, 2/6, 4, and 5, but not TLR 7/8 in the index patient. We previously found that TLR2/6 and TLR4 can interact with RSV F to promote innate immune responses [7, 8]. It is uncertain which TLR or comolecules are required for CD14-RSV F signaling in our patient, but direct interaction between CD14 and RSV F protein, facilitated by the presence of MD2, has been reported [8, 9, 40]. Dysregulation of TLR signaling impacts various functions within the immune system, including negative regulation of dendritic cell maturation and changing B-cell proliferation [41]. Defective TLR signaling has been associated with enhanced RSV disease caused by poor antibody affinity maturation [42]. In our patient, antibody affinity, avidity, and B-cell memory were normal, suggesting intact antibody maturation in CD14 deficiency.

We were surprised to find that our patient did not present with invasive bacterial infections. In vivo trials with CD14 knock out mice have shown a protective effect of CD14 by attenuating bacterial growth and dampening the systemic inflammatory response against Gram-negative bacterial infection [43]. In humans, in Phase 1 trials, the treatment with a chimeric CD14 antibody (IC14) showed to protect to LPS induced systemic inflammatory response, and it did not increase the incidence of secondary infection [44]. These data, taken together, suggest a protective role of CD14 during bacterial-induced systemic inflammation and could explain the low burden of severe bacterial infections in our patient.

A strength of our study is that we were able to perform extensive clinical, genetic, epithelial, and immunological phenotyping. We were able to address part of the longstanding question about the clinical relevance of CD14 in RSV disease. Furthermore, the broad clinical phenotype of recurrent respiratory infections, and our in vitro TLR data, indicated a central role for CD14 in innate immune signaling that extends beyond TLR4. Soluble CD14 is studied as a marker for severe disease during viral infections, including in individuals with human immunodeficiency virus infection [45]. Therapeutics targeting CD14 have been studied and are currently being developed, such as the use of a monoclonal anti-CD14 antibody (IC14) for the treatment of severe COVID-19 [37, 46]. Our study exposes potential adverse effects of such therapies.

Limitations also require discussion. First, we described a single CD14-deficient patient, and no other patients could be identified because of the low allele frequency. However, there are several other loss of function mutations identified in the CD14 gene (combined allele frequency of 2.5 × 10^4^), although no other CD14-deficient patient has been described thus far. Second, the amount of blood limited the number of tests we could do. For instance, we did not investigate phagocytosis or monocyte responses to viruses other than RSV. Third, for practical reasons, adult controls were used for stimulation experiments with immune cells. We found impaired TLR 1/2, 2/6, 4, and 5 responses in the index patient up to age 3, when TLR responses are at adult level in normal children [47, 48]. For the epithelial studies, we were able to use healthy pediatric controls. Finally, we only had access to samples of the upper airway of the CD14-deficient patient. Growth kinetics of RSV and viral titers are higher in bronchial compared with nasal cell cultures. In addition, immune responses in the upper and lower airway tract show regional differences [49]. However, both culture models show similar proinflammatory responses to RSV infection and therefore are a suitable model to study RSV infection in vitro [50].

## CONCLUSIONS

We described a novel single-gene immunodeficiency resulting in a phenotype characterized by, but not limited to, recurrent RSV infections. This patient establishes an important role for CD14 in RSV pathogenesis. Furthermore, CD14-mediated innate immune responses are likely involved in the immune response against pathogens in the respiratory tract. We predict that other loss of function mutations in the CD14 signaling pathway may be similarly associated with recurrent or severe RSV infections.

## Supplementary Data

Supplementary materials are available at *The Journal of Infectious Diseases* online. Supplementary materials consist of data provided by the author that are published to benefit the reader. The posted materials are not copyedited. The contents of all supplementary data are the sole responsibility of the authors. Questions or messages regarding errors should be addressed to the author.

jiac114_suppl_Supplementary_Figure_S1

jiac114_suppl_Supplementary_Figure_S2

jiac114_suppl_Supplementary_Figure_S3

jiac114_suppl_Supplementary_Figure_S4

jiac114_suppl_Supplementary_Figure_S5

jiac114_suppl_Supplementary_Figure_S6

jiac114_suppl_Supplementary_Figure_S7

jiac114_suppl_Supplementary_Figure_S8

jiac114_suppl_Supplementary_Figure_S9

jiac114_suppl_Supplementary_Figure_S10

jiac114_suppl_Supplementary_Figure_S11

jiac114_suppl_Supplementary_Figure_S12

jiac114_suppl_Supplementary_Material

## Notes

***Acknowledgments.*** We are most thankful to the patients and parents/caregivers for participating in this study. We acknowledge Samuel Wright for his knowledge and advice. We thank Michiel van der Vlist, Inês Ramos, Margreet Westerlaken, and Akashdip Singh for technical support. We thank Tom Wolfs for critically reviewing the manuscript.

***Author contributions.*** S. B. B., L. J. B., and L. M. contributed to conceptualization. S. B. B., L. J. B., L. M., L.W.R., G. D. A., E. A. K.-J., D. G., and B. S. G. contributed to methodology. S. B. B., E. P., H. H. M. R., R. v. S., M. R., J. C., R. M. S., S. G. S., K. D., L. A. C., S. N., K. M. M., T. J. R., and T. v. d. B. contributed to experiments. S. B. B. and N. V. contributed to visualization. L. J. B., E. A. K.-J., and D. G. contributed to funding acquisition. N. D. contributed to project administration. L. J. B., L. M., G. v. H., J. M. B., G. D. A., G. A. M. B., E. A. K.-J., D. G., and B. S. G. contributed to supervision. S. B. B. and L. J. B. contributed to writing the original draft. S. B. B., A. P., M. R., N. V., G. D. A., and L. J. B. contributed to writing, review, and editing. All authors were involved in writing the paper and had final approval of the submitted and published versions.

***Financial support.*** This work was funded by an internal University Medical Centre Utrecht Grant (to L. J. B.). D. G. and E. A. K.-J. report grants from National Institute of Health. The rest of the authors declare that they have no relevant conflicts of interest.

***Potential conflicts of interest*.** All authors: No reported conflicts of interest. All authors have submitted the ICMJE Form for Disclosure of Potential Conflicts of Interest.
