# Supplementary figures and images for: Recurrent Respiratory Syncytial Virus Infection in a CD14-Deficient Patient
Source: J Infect Dis. 2022 Apr 16;226(2):258–69. doi: 10.1093/infdis/jiac114 (PMC9400420; doi:10.1093/infdis/jiac114)

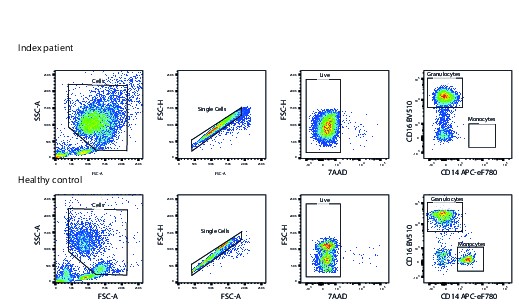

Supplement: jiac114_suppl_Supplementary_Figure_S1 [file jiac114_suppl_supplementary_figure_s1.jpeg]

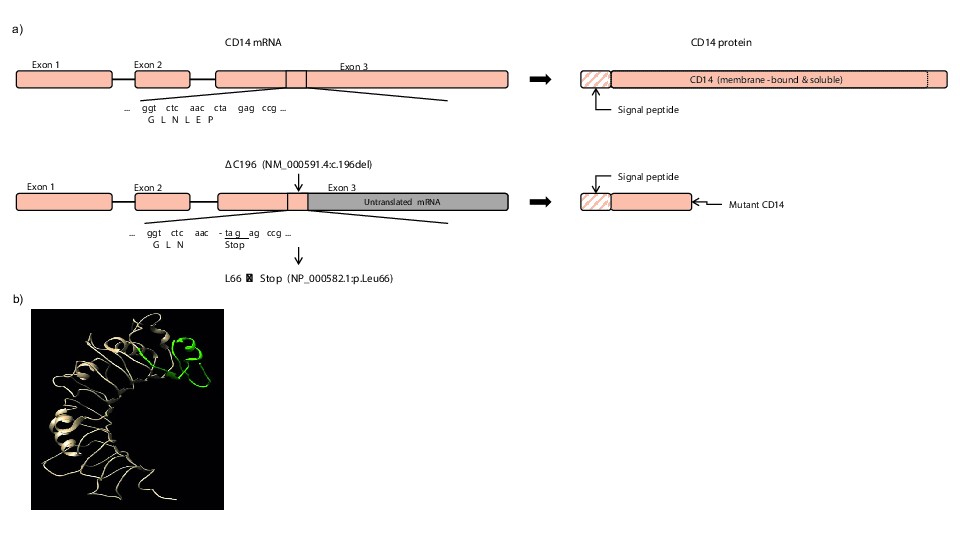

Supplement: jiac114_suppl_Supplementary_Figure_S2 [file jiac114_suppl_supplementary_figure_s2.jpeg]

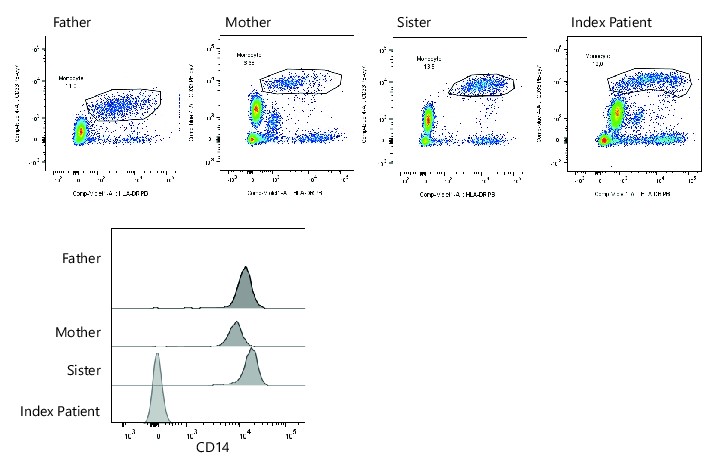

Supplement: jiac114_suppl_Supplementary_Figure_S3 [file jiac114_suppl_supplementary_figure_s3.jpeg]

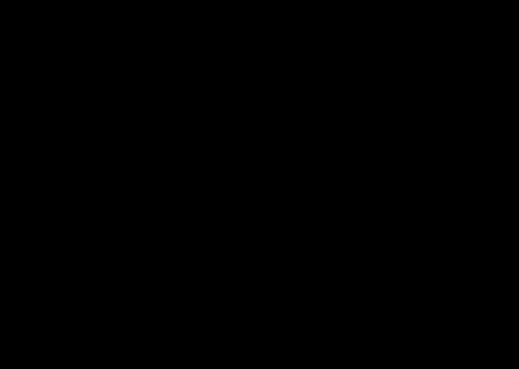

Supplement: jiac114_suppl_Supplementary_Figure_S4 [file jiac114_suppl_supplementary_figure_s4.jpeg]

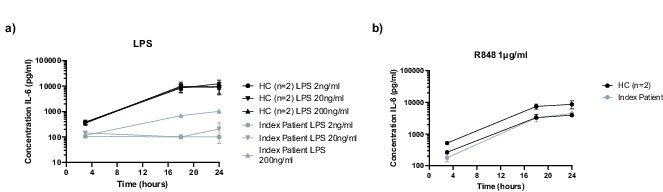

Supplement: jiac114_suppl_Supplementary_Figure_S5 [file jiac114_suppl_supplementary_figure_s5.jpeg]

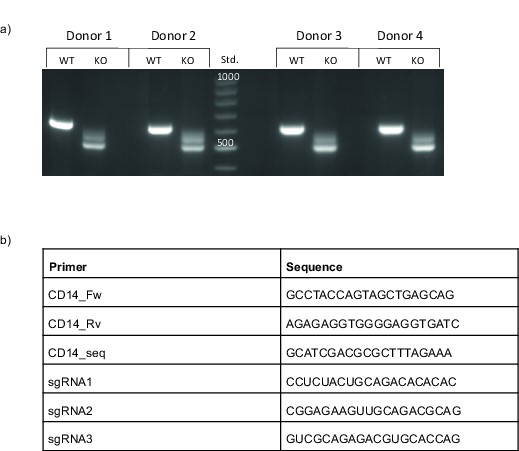

Supplement: jiac114_suppl_Supplementary_Figure_S6 [file jiac114_suppl_supplementary_figure_s6.jpeg]

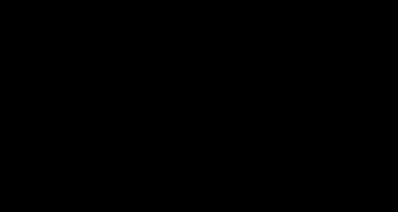

Supplement: jiac114_suppl_Supplementary_Figure_S7 [file jiac114_suppl_supplementary_figure_s7.jpeg]

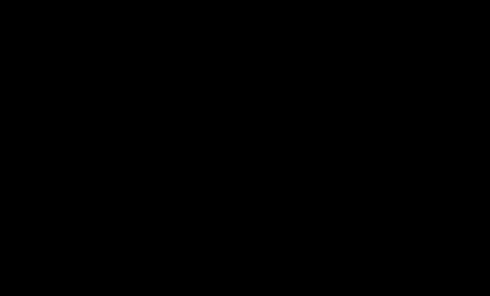

Supplement: jiac114_suppl_Supplementary_Figure_S8 [file jiac114_suppl_supplementary_figure_s8.jpeg]

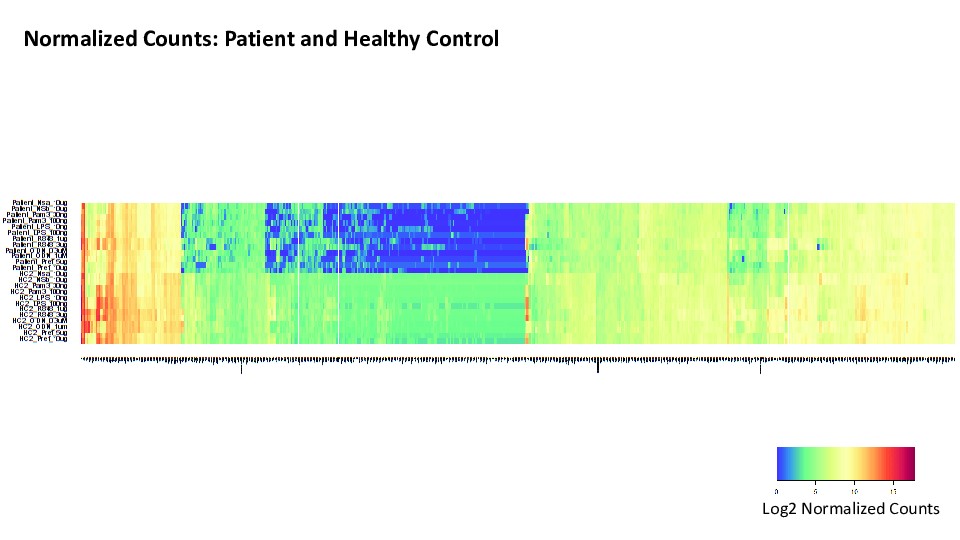

Supplement: jiac114_suppl_Supplementary_Figure_S9 [file jiac114_suppl_supplementary_figure_s9.jpeg]

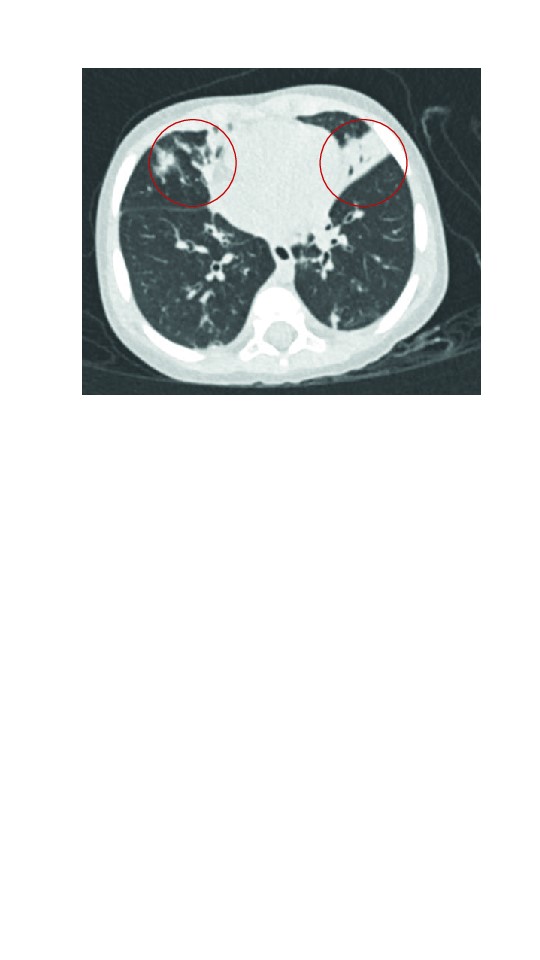

Supplement: jiac114_suppl_Supplementary_Figure_S10 [file jiac114_suppl_supplementary_figure_s10.jpeg]

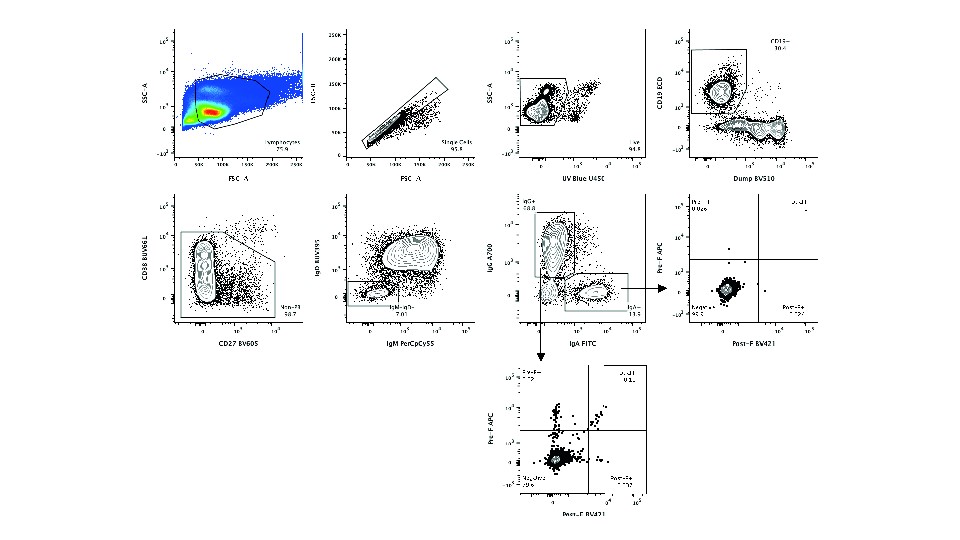

Supplement: jiac114_suppl_Supplementary_Figure_S11 [file jiac114_suppl_supplementary_figure_s11.jpeg]

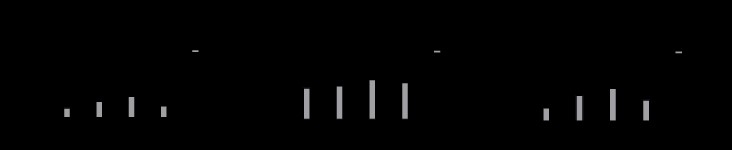

Supplement: jiac114_suppl_Supplementary_Figure_S12 [file jiac114_suppl_supplementary_figure_s12.jpeg]
