# Supplementary material for: Recurrent Respiratory Syncytial Virus Infection in a CD14-Deficient Patient
Source: J Infect Dis. 2022 Apr 16;226(2):258–69. doi: 10.1093/infdis/jiac114 (PMC9400420; doi:10.1093/infdis/jiac114)
Supplement: jiac114_suppl_Supplementary_Material [file jiac114_suppl_supplementary_material.docx]

Online supplement

**Recurrent respiratory syncytial virus infection in a CD14 deficient patient Case description**

The index patient was the second child of two non-consanguineous parents. He was born at term with a birthweight of 4470 grams. He exhibited normal growth and reached developmental milestones at the appropriate age. At the age of six months, he presented at the outpatient clinic with mild viral wheeze and started with salbutamol inhalations for occasional use. There were no signs of eczema or allergy. He was regularly immunized according to the national immunization program. The family history was negative for atopy, immuno-deficiencies or gastrointestinal diseases. Although both parents had the same ethnic background, their ancestors came from different regions. Therefore, we cannot confirm consanguinity.

At the age of nine months, the patient presented in the emergency department with vomiting, diarrhea and minimal fecal blood loss. He was diagnosed with mild viral gastro-enteritis, without signs of dehydration, and sent home. There was no pathogen identified. One week later, he presented again in the emergency department. The enteral complaints subsided, but he presented with a fever of 39°C, dyspnea and wheezing. He was admitted to the pediatric ward for monitoring and supplemental oxygen support through a high flow nasal cannula. Due to progressive dyspnea, he required intubation for invasive mechanical ventilation in the pediatric intensive care unit (PICU). At the PICU, he was diagnosed with RSV bronchiolitis with a bacterial superinfection of beta-lactamase positive *Haemophilus influenzae*. He was prescribed a seven day course of amoxicillin and clavulanic acid. However, two days after the discontinuation of the antibiotic therapy, he became increasingly ill with fever of 40.1 °C, a rise in CRP levels to a maximum of 205 mg/L and deteriorating pulmonary status leading to the need of high ventilator pressure. Sputum cultures showed a resurgence of *H. influenzae* growth, and urine cultures revealed the growth of *E. coli* and *Klebsiella pneumoniae*. Blood cultures were negative. The patient was put on ceftazidime intravenously for seven days. Because of anemia with a hemoglobin level 4.4mmol/L, he received packed cells. During admission, he developed mild edema, which was treated with furosemide and spironolactone. Sedation medication consisted of intravenous midazolam and morphine. He was mechanically ventilated for 11 days and discharged the next day to the pediatric ward. In the ward, he required supplemental oxygen through a low flow nasal cannula for 14 days. Furthermore, the patient developed a delirium, due to withdrawal of sedatives, which was treated with risperidone and methadone. After 15 days in the pediatric ward, he was discharged home.

During the admission to the PICU, the patient was enrolled in a study regarding neutrophil function during RSV bronchiolitis.[1] In this study, we found absence of the CD14 receptor on the patient’s monocytes by flow cytometry (supplementary figure S1). Neutrophil function - oxidative burst by sputum neutrophils and neutrophil extracellular trap-formation by blood neutrophils - were comparable to other infants with severe RSV. The leukocyte differential count was normal, with average monocyte counts. We excluded paroxysmal nocturnal hemoglobinuria as a cause of absent CD14, since other GPI-anchored receptors were present (FLAER, CD157 and CD59). Finally, Sanger sequencing of the CD14 gene revealed a single-nucleotide deletion in the CD14 gene: a homozygous variant (NM_000591.4:c.196del), resulting in a frameshift and truncation in the CD14 protein (NP_000582.1:p.Leu66*) (figure 1, C and supplementary figure S2). Both parents were heterozygous for the same mutation, with normal CD14 expression on monocytes (supplementary figure S3). This confirmed the diagnosis of an autosomal recessive (AR) CD14 deficiency.

In the subsequent months, the index patient developed frequent episodes of otitis media with otorrhea, treated with antibiotic auricular drops (containing dexamethasone, framycetin and gramicidin), and recurrent cervical lymphadenitis. At the age of 13 months, he was prescribed antibiotic prophylaxis with [trimethoprim-sulfamethoxazole.](https://www.ncbi.nlm.nih.gov/pubmed/30000348) He continued to use salbutamol inhalations on occasion, and at the age of 2.5 years he was prescribed corticosteroid inhalation therapy. His weight and height gain were normal.

At the age of 14 and 22 months and 4 years, he was again admitted to the pediatric ward with respiratory distress due to RSV bronchiolitis. At age 14 months, he received no supplemental oxygen and was released after one day. At 22 months and age 4 years, he required a low flow nasal cannula for respiratory support over a period of four and two days respectively. Additionally, he was admitted three times with acute respiratory tract infection (ARTI) and wheezing due to other viral pathogens in the first four years of life. At the age of 21 months we explored microbial flora in the airways of the patient, this showed normal commensal flora. Table S2 and S3 show an overview of all virology and bacteriology results.

To evaluate the possibility of permanent lung damage as a result of recurrent RTI’s and CD14 deficiency, we performed a CT scan of the lungs at the age of 21 months. The CT scan showed mild ground glass phenomena surrounding the apex and in the right and left lower lobe (supplementary figure S10). IgE was modestly elevated (176 kU/L) and showed no specific IgE against respiratory allergens. Despite incidental episodes of viral wheeze, he is not limited in his daily activities due to pulmonary complaints. At the age of four we performed lung function measurement which was technically challenging because of the child’s age. We will repeat this when the patient is older. Additional immunological investigation did not reveal abnormalities in other immune cell functions (normal B and T cell subsets, IgA, IgM and IgG, and a normal response to 10-valent pneumococcal vaccination) (table S4 and S5). Broad viral serology was negative for EBV, influenza A and B, and positive for CMV, and VZV. Vaccination responses to polio, measles, diphtheria and tetanus were normal. Mumps vaccination failed to elicit a protective response after the first immunization at age 14 months (table S6). A SNP-array showed no abnormalities. Whole exome sequencing showed, besides the CD14 mutation, a heterozygous mutation in the ABCB4 gene. This is rarely associated with progressive familial intrahepatic cholestasis (#OMIM 602347), but liver enzymes tested normal in our patient. No other de novo or recessive variants were identified that score as variant of uncertain significance or higher according to the 2015 ACMG-AMP guidelines.*[2]*

**Supplementary methods**

**Patient and samples**

The patient and infant controls were initially enrolled through a study previously performed by our group and published elsewhere (Neon Study: IRB review reference number NL58404.041.16).[1] All participants, or the legal guardian in the case of a minor, gave written informed consent. Healthy adult donors were used when indicated. For the index patient, all procedures were based on standard of care and established clinical guidelines were followed. The Parent Advisory Board of our RSV research team was involved in writing a patient information letter specifically for the index patient.

Collection of blood and plasma samples of the control children were within 48 hours after admission to the PICU. Nasopharyngeal swabs for determination of the RSV subtype and Ct value were taken within 48 hours after admission to the PICU. Severe RSV was defined as being in the need for mechanical ventilation at the PICU.

**Sanger sequencing**

Sanger sequencing confirmed CD14 deficiency. Specifically, CD14 coding exons were amplified and sequenced with gene specific primers (primer sequences and cycling conditions available upon request).

**PBMC and monocyte isolation and stimulation**

Peripheral blood mononuclear cells (PBMCs) were extracted from whole blood using Ficoll. First, the blood was diluted 1:1 with PBS, added on top of Ficoll and centrifuged for 30 minutes, 1000 RCF, acceleration 2, break 2, at room temperature. PBMCs were collected and washed twice. Monocytes were isolated from the PBMCs by Percoll gradient. Standard Isotone Percoll (SIP) was prepared by adding 10:1 10x PBS. SIP was then diluted to a concentration of 34%, 47.5% and 60% with IMDM containing 1% FCS. PBMCs were taken up in 60% SIP, followed by gentle addition of 47.5% and 34% SIP. This was centrifuged for 45 minutes, 1750 RCF, acceleration 2, break 2, at room temperature. Monocytes were collected from the upper ring and washed twice with PBS. A sample was taken to determine monocyte purity by flow cytometry.

After isolation, PBMCs or monocytes were counted and transferred to a plate: 100.000 cells per well in a 96 well plate, 500.000 cells per well in a 24 well plate or 1.000.000 cells per well in a 12 well plate. They were stimulated with a panel of Toll Like receptor (TLR) agonists: LPS (S. typhosa (Sigma Aldrich), or E. coli Ultra pure) 1, 10 and 100 ng/mL (TLR4), Lipoteichoic acid 0.1 and 1µg/mL (TLR2 (Sigma Aldrich)), PAM3CSK 30 and 100 ng/mL (TLR2/6), FSL-1 1 and 10 µ/mL (TLR2/6), Poly I:C 0.1 and 1 µg/mL (TLR3), flagellin 1 and 5 µg/mL (TLR5), R848 1 or 3 µg/mL (TLR7 and TLR8), ODN 0.3 and 1 mM (TLR9). All TLR stimuli were obtained from Invivogen, unless stated otherwise. Where indicated monocytes were pre-incubated for 30 minutes at 4°C with 10µg/mL anti-hCD14-IgG (Invivogen) or mouse anti-human HLA-ABC (BD Pharmingen) as control. Polymyxin B 10µg/mL (Invivogen) was added to the PBMCs 10 minutes prior to the addition of LPS or RSV F. For heat inactivation, LPS and RSV F were heated during 10 min at 99°C before adding this to the PBMCs. Supernatant was collected at the designated time points, and cells were lysed using RLT plus (Qiagen). Samples were stored at -80°C degrees until further processing. All assays with cells derived from the index patient were performed within the age range of 10 months to 3 years.

**Flow Cytometry**

We performed flow cytometry to identify CD14 surface expression on patient and control immune cells. The whole blood sample was depleted from red blood cells, by lysing the erythrocytes by incubation of 45 second with 5x the initial blood volume of cold distilled water, after which 0.1x volume of 10x PBS (=1 mL) was added to halt cell lysis. Cells were washed and remaining erythrocytes were removed by a second lysis with ammonium chloride buffer for 5 - 10 minutes, and centrifuged at 500g for 5 min at 4°C. The remaining cell pellet was washed with RPMI-1640 medium supplemented with 10% heat-inactivated Fetal Calf serum (FCS). Cell were stained for surface markers for 20 min at 4°C in PBS containing 0.01% (m/v) sodium azide and 1% (m/v) bovine serum albumin (BSA). Flow cytometric analysis was performed using a CANTO II (BD) and analyzed with FlowJo 10.2 software (FlowJo LLC). Cell types were identified according to their characteristic forward and side light-scatter properties, and by their typical cell surface markers: neutrophils: CD14^-^/CD16^+^, monocytes: CD14^+^/CD16^-^. 7-aminoactinomycin D (7-AAD) was used to distinguish apoptotic cells. Absence of CD14 surface expression in patient samples was demonstrated by flow cytometry using two different clones (aCD14 clones RMO52 and 61D3). Clone 61D3 has been shown to compete for binding to CD14 with aCD14 clone MEM18[3], which has been mapped to amino acids 51 to 63 of CD14 protein[4]. Both clones (61D3 and MEM18) act also as functional blockers of CD14: macrophages pre-incubated with either clone fail to produce TNF in response to LPS.[4] We are therefore confident that we are using an anti-CD14 clone that is capable of detecting also the truncated mutant version of CD14.

To evaluate monocyte purity after Percoll, monocyte subtypes were identified as follows: lymphocytes were selected based on their forward and side scatter properties, single cells were selected, leukocytes were identified by CD45 surface expression. Monocyte subtypes were identified based on CD16 and CD14 surface expression. Non-classical monocytes: CD16^+^/CD14^-^, Intermediate monocytes: CD16^+^/CD14^+^, Classical monocytes: CD16^-^/CD14^-^. The mean monocyte purity after Percoll isolation was 65%.

The generation of RSV-pre and post-F probes and identification of RSV-pre and post-F binding B-cell was performed as described previously.[5] Briefly, frozen PBMCs were thawed and stained for viability with the Fixable Blue Dead Cell Stain Kit (Thermofisher Scientific), and then surface stained with the B cell antibody panel in table S1. Samples were collected on a LSR-X50 cytometer (BD) and data were analyzed with FlowJo 10.2 software. Table S1 shows a list of reagents and dilutions used in flow cytometry studies. The gating strategy is depicted in supplementary figure S11.

**mRNA expression**

Extracted mRNA (RNeasy Mini Kit Qiagen) was used for cDNA synthesis, using iScript.

NanoString analysis was performed using the nCounter SPRINT profiler, using the immunology_v2_C2328 Codeset, which simultaneously measures mRNA expression levels of 579 inflammation-related genes, according to the manufacturer’s instructions (NanoString Technologies^TM^).

**ELISA and quantification of RSV F protein-binding antibody**

Standard ELISA kits were used according to the manufacturers’ protocols to measure: IL-6 and IL-8 (Ready-Set-Go! (V2) Ebioscience), CD14 and LBP (both RnD systems). Serum binding assays were performed as described previously.[5]

**RSV IgG concentrations and avidity assays**

Analysis of IgG in serum was performed using a RSV multiplex immunoassay as described earlier.[6] The IgG concentrations against five RSV proteins (Ga, Gb, prefusion F, postfusion F and nucleoprotein) were quantified by interpolation from a five-parameter logistic standard curve, converting mean fluorescence intensities (MFI) to arbitrary units per ml (AU/mL).

The avidity of the RSV-specific antibodies was determined using the same RSV multiplex immunoassay with some adaptations. After incubation of the sera with the RSV proteins, all samples were additionally incubated for 10 minutes, at room temperature, in threefold on the same plate in the presence of 1.5 M Ammonium thiocyanate (NH_4_SCN), 3.0 M NH_4_SCN or PBS (pH 7.4). The avidity index (AI) was expressed as the percentage of residual MFI IgG signal in comparison to the undenatured (PBS) signal which was set at 100%.

**RSV neutralization assays**

Neutralization by sera from the index patient, 15 adult controls of a previous study (reference standard)[7], and the BEI NR-4020 control sera were measured by a fluorescence plate reader neutralization assay described previously.[5] In brief, diluted sera were mixed with an equal volume of recombinant mKate-RSV expressing prototypic F genes from subtype A (strain A2) or subtype B (strain 18537), and incubated at 37°C for 1 hour. Next, the mixture was added to H28 cells that had been seeded in a 384-well black optical bottom plate. After 24 hours of incubation plates were analyzed on a spectrophotometer (588/635 nm). Neutralization assays used to address the contribution of pre-F -exclusive antibodies were performed by dilution of sera in post-F protein from RSV A2 (20 µg/mL) prior to the addition of the virus.

**Collection and differentiation of nasal epithelial cells**

Primary human nasal epithelial cells (HNECs) were obtained from healthy children, and the index patient by nasal brushes. All participants or their caregivers provided written informed consent. The study was approved by a specific ethical board for the use of biobanked materials TcBIO (Toetsingscommissie Biobanks), an institutional Medical Research Ethics Committee of the University Medical Center Utrecht (protocol ID: 19/678). Nasal airway cells were collected, isolated, expanded and differentiated as previously described.[18] The basal cells were differentiated on Transwell® inserts (Corning) in an air-liquid interface (ALI). They were cultured for ~18 days to obtain a complete differentiated epithelium. Culture medium was refreshed twice a week, including an apical wash of 120 µl PBS (5 minutes incubation).

**Generation of CD14 knockouts in airway epithelial basal cells using CRISPR-Cas9**

CD14 knock-out cell lines were made with CRISPR-Cas9 technologies as described previously.[19, 20] Before electroporation, sgRNA (30 µM, Synthego) and Cas9 protein (20 µM, Synthego) were mixed with optiMEM supplemented with Y27632 (10 µM) and incubated for 10 minutes at room temperature to generate RNP complexes. One million trypsinized basal cells per sample were dissolved in 75 µL of optiMEM supplemented with Y27632 (10 µM) and added to the RNP complex mixture. The NEPA21 electroporator was used for transfection according to previously published settings [21]. After electroporation the basal cells were expanded. 0.2 million cells were used for DNA isolation and the other cells were seeded for ALI differentiation. DNA was isolated according to the manual of the Quick-DNA Microprep Kit (Zymo research). Regions of interest were amplified in a PCR reaction with GoTaq G2 Flexi DNA polymerase with primers, and PCR-amplified samples were run on 1,2% TBE-agarose gel for size separation. DNA fragments were excised from the gel, purified according to the gel extraction kit (Qiagen), and sent for Sanger sequencing with sequencing primers (supplementary figure S5).

To compare the epithelial cell differentiation between patient derived WT and CD14 human nasal epithelial cell cultured at an air liquid interface (HNEC-ALI), we measured the expression of MUC5AC (goblet cells), FOXJ1 (ciliated cells) and P63 (basal cells) by qPCR. There were no differences in gene expression between WT and CD14-/- cultures (Supplementary figure S12). For RNA isolation, cells were lysed using RLT + 1% β-mercaptoethanol. Extracted mRNA (RNeasy Mini Kit Qiagen) was used for cDNA synthesis, using iScript. For relative gene expression, cDNA transcripts were quantified by real-time quantitative PCR with IQTM SYBR Green Supermix (Bio-Rad, FR) and specific primers. Primers used: B-actin (Fw CTGGAACGGTGAAGGTGACA, Rv AAGGGACTTCCTGTAACAATGCA) and RPL13A (Fw CCTGGAGGAGAAGAGGAAAGAGA, Rv TTGAGGACCTCTGTGTATTTGTCAA) (household genes), CD14 (Fw GAACCTTGTGAGCTGGACGA, Rv CAGACACACACTGGAAGGCT), MUC5AC (Fw ATTTTTTCCCCACTCCTGATG, Rv AAGACAACCCACTCCCAACC), FOXJ1 (Fw GGAGGGGACGTAAATCCCTA, Rv TTGGTCCCAGTACCAGC), P63 (Fw CCA CCTGGACGTAT CCACTG, Rv TCGAATCAAATGACTAGGGG).

**Viral infection of HNEC-ALI**

One day before infection, culture medium of the HNEC-ALI was refreshed. RSV-A2-GFP was diluted in serum-free Opti-MEM (Gibco) to obtain a multiplicity of infection (MOI) of 1. HNEC-ALI were infected at the apical side with 100 μl RSV-A2-GFP or serum-free Opti-MEM only (mock), and incubated at 37°C, 5% CO2 for two hours. After incubation, apical medium was aspirated carefully and the apical surface was washed twice with 125μl of warm PBS. After infection the cultures were incubated for up to 72 hours. Each day, basal medium was refreshed and stored, without washing the apical compartment. Pictures were taken each day with an EVOS microscope using a 40x and 100x objective. At the indicated day post infection (dpi), 200μl of serum-free Opti-MEM was added to the inserts and incubated for 30 minutes at 37°C, 5% CO2. Supernatant from both the apical and basal compartment were stored for ELISA. Subsequently, cultures were lysed for gene expression by real-time quantitative PCR. From the apical wash, viral titers were measured by the Rijksinstituut voor Volksgezondheid en Milieu (RIVM).

**RNA isolation and real-time quantitative PCR analysis of ALI**

For RNA isolation, cells were lysed using RLT + 1% β-mercaptoethanol. Biological duplicate lysates were pooled to obtain a final cDNA concentration of 500ng/µl. Extracted mRNA (RNeasy Mini Kit QIAGEN) was used for cDNA synthesis, using iScript (Bio-Rad) and a T100 Thermal Cycler machine (Bio-Rad).

For relative gene expression, cDNA transcripts were quantified by real-time quantitative PCR (CFX Connect Real time PCR, CFX-96 Real time PCR Bio-Rad) with IQTM SYBR Green Supermix (Bio-Rad, FR) and specific primers for RPL13A, B-ACTIN, P63, MUC5AC, FOXJ1, and CD14. Expression levels of evaluated genes were calculated by relative quantification, using the standard curve method. Additionally, each value was corrected for the expression of the following housekeeping genes: B-ACTIN or RPL13A.

**References**

1. Besteman SB, Callaghan A, Hennus MP, Westerlaken GHA, Meyaard L, Bont LL. Signal inhibitory receptor on leukocytes (SIRL)-1 and leukocyte- associated immunoglobulin-like receptor (LAIR)-1 regulate neutrophil function in infants. Clin Immunol **2020**; 211:108324.

2. Richards S, Aziz N, Bale S, et al. Standards and guidelines for the interpretation of sequence variants: a joint consensus recommendation of the American College of Medical Genetics and Genomics and the Association for Molecular Pathology. Genet Med **2015**; 17:405-24.

3. Devitt A, Moffatt OD, Raykundalia C, Capra JD, Simmons DL, Gregory CD. Human CD14 mediates recognition and phagocytosis of apoptotic cells. Nature **1998**; 392:505-9.

4. Kelley SL, Lukk T, Nair SK, Tapping RI. The crystal structure of human soluble CD14 reveals a bent solenoid with a hydrophobic amino-terminal pocket. J Immunol **2013**; 190:1304-11.

5. Crank MC, Ruckwardt TJ, Chen M, et al. A proof of concept for structure-based vaccine design targeting RSV in humans. Science **2019**; 365:505-9.

6. Schepp RM, de Haan CAM, Wilkins D, et al. Development and Standardization of a High-Throughput Multiplex Immunoassay for the Simultaneous Quantification of Specific Antibodies to Five Respiratory Syncytial Virus Proteins. mSphere **2019**; 4.

7. Phung E, Chang LA, Morabito KM, et al. Epitope-Specific Serological Assays for RSV: Conformation Matters. Vaccines (Basel) **2019**; 7.

**Supplementary tables**

**Table S1. Overview of antibodies used for flow cytometry**

| **Panel used in figure 1a** | | | | |
| --- | --- | --- | --- | --- |
| **Target** | **Label** | **Clone** | **Company** | **Dilution** |
| CD16 | PeCy7 | 3G8 | BioLegend | 100x |
| CD14 | APC-eF780 | 61D3 | eBioscience | 200x |
| 7-aminoactinomycin D (7-AAD) |  |  | BD Bioscience | 5 µl/sample |
| **Panel used to determine monocyte purity** | | | | |
| **Target** | **Label** | **Clone** | **Company** | **Dilution** |
| CD14 | V450 | G10F5 | BD Bioscience | 50x |
| CD16 | PeCy7 | 3G8 | BioLegend | 100x |
| CD45 | APC-eF780 | 61D3 | eBioscience | 200x |
| **Panel used to determine monocyte subsets** | | | | |
| **Target** | **Label** | **Clone** | **Company** | **Dilution** |
| CD3 | PerCP-Cy5.5 | UCHT1 | Biolegend | 100x |
| CD19 | PerCP-Cy5.5 | HIB19 | eBioscience | 100x |
| CD56 | PerCP-Cy5.5 | B159 | BD | 100x |
| CD66b | PerCP-Cy5.5 | G10F5 | Biolegend | 100x |
| CD14 | APC-eF780 | 61D3 | eBioscience | 200x |
| CD16 | V500 | 3G8 | BD | 50x |
| CD86 | FITC | 2331 (FUN-1) | BD | 50x |
| CD33 | PE | WM53 | Biorad | 20x |
| CD64 | APC | 10.1 | Biolegend | 20x |
| CCR2 | BV421 | K036C2 | Biolegend | 150x |
| HLA-DR | PE-Cy7 | L243 | Biolegend | 100x |
| **Panel used to quantify RSV F-specific B-cells** | | | | |
| **Target** | **Label** | **Clone** | **Company** | **Volume used per 100 µl test** |
| IgA | FITC | S11-8E10 | Miltenyi | 2.5 µl |
| IgM | PerCpCy55 | G20-127 | BD | 5 µl |
| CD8 | BV510 | RPA-T8 | Biolegend | 0.5 µl |
| CD3 | BV510 | OKT3 | Biolegend | 0.625 µl |
| CD56 | BV510 | HCD56 | Biolegend | 0.625 µl |
| CD14 | BV510 | M5E2 | Biolegend | 0.31 µl |
| CD27 | BV605 | O323 | Biolegend | 2 µl |
| CD11c | BV650 | 3.9 | Biolegend | 0.25 µl |
| CD72 | BV711 | J4-117 | BD Bioscience | 0.15 µl |
| CD19 | ECD | J3-119 | Beckman | 2.5 µl |
| CD21 | PeCy5 | B-ly4 | BD Bioscience | 2.5 µl |
| CD71 | PeCy7 | CY1G4 | Biolegend | 0.078 µl |
| IgD | BUV395 | IA6-2 | BD Bioscience | 1 µl |
| UV Blue |  |  | Thermofisher | 0.125 µl |
| CD38 | BUV661 | HIT2 | BD Bioscience | 1.25 µl |
| IgG | A700 | G18-145 | BD Bioscience | 2.5 µl |
| CD20 | APCCy7 | 2H7 | Biolegend | 0.5 µl |
| Pre-F Probe | APC |  | In-House Conjugate | 0.24 µg |
| Post-F Probe | BV421 |  | In-House Conjugate | 0.24 µg |

**Table S2. Overview of virology test results**

| **Age (months)** | **Material** | **Method** | **Result** |
| --- | --- | --- | --- |
| **9** | NP swab | PCR | Boca virus & RSV B positive |
| **11** | NP swab | PCR | Boca virus & RSV negative |
| **14** | NP swab | FARP | RSV B positive |
| **15** | NP swab | FARP | Rhino virus positive, RSV negative |
| **18** | NP swab | PCR | Parainfluenza virus, type 1 & 3, & Adenovirus positive |
| **21** | NP swab | ePlex | rhinovirus/enterovirus & coronavirus positive |
| **22** | NP swab | ePlex | RSV A positive, rhinovirus/enterovirus & coronavirus negative |
| **22** | NP swab | PCR | RSV negative |
| **25** | NP swab | ePlex | rhinovirus/enterovirus positive |
| **28** | NP swab | ePlex | rhinovirus/enterovirus negative |
| **29** | NP swab | ePlex | rhinovirus/enterovirus positive |
| **29** | NP swab | ePlex | rhinovirus/enterovirus positive |
| **31** | feces | PCR | adenovirus positive |
| **32** | feces | PCR | adenovirus negative |
| **33** | NP swab | ePlex | rhinovirus/enterovirus positive |

NP = nasopharyngeal, PCR = polymerase chain reaction, FARP = FilmArray respiratory panel, Eplex = multiplex molecular based assay.

**Table S3. Overview of bacterial culture results**

| **Age (months)** | **Culture** | **Microorganism** |
| --- | --- | --- |
| **9** | urine (from indwelling catheter) | negative |
|  | blood culture | negative |
|  | bronchotracheal aspirate | *H. influenzae* |
| **10** | bronchotracheal aspirate | *H. influenzae* |
|  | catheter urine | *Klebsiella pneumoniae* |
|  |  | *E. coli* |
|  | blood culture | negative |
| **13** | ear swab | *H. influenzae* |
| **20** | ear swab (right ear) | *Streptococcus pyogenes* |
|  | ear swab (left ear) | *Streptococcus pyogenes* |
| **21** | ear swab | negative |
| **21** | bronchotracheal aspirate* | *Streptococcus mitis* group |
|  |  | *Rothia mucilaginosa* |
|  |  | *Neisseria subflava* |
|  |  | *Prevotella* spp, |

* To explore the microbial flora in the airways of the patient, sputum bronchotracheal aspirate was obtained at the age of 21 months and cultured using routine media and additional media (anaerobic culture and yeast/mold culture). All visually discernible microorganisms were identified by MALDI-TOF-MS. The identified microorganisms fit with normal commensal flora from the oropharynx and the upper airways. No yeasts or molds were isolated. All other cultures were performed in relation to upper respiratory tract complaints with routine procedures only.

**Table S4. Normal immune phenotyping and immunoglobulin levels**

| **Immune Phenotype** | | | |
| --- | --- | --- | --- |
| **Test** | **Result** | **Reference** | **Unit** |
| **Relative T-cell** | 53 | 53-74 | % in Ly |
| **Absolute T-cell** | 2,630 | 1800-5900 | per mm^3 |
| **Relative CD4** | 25.40 | 33.0-55.0 | % in Ly |
| **Absolute CD4** | 1,267 | 1902-2977 | per mm^3 |
| **Relative CD8** | 24 | 14.0-26.0 | % in Ly |
| **Absolute CD8** | 1,197 | 667-1473 | per mm^3 |
| **CD4/CD8 ratio** | 1.10 | 1.4-3.7 |  |
| **Active T cell (CD4)** | 1.70 | 0.3-1.3 | % in CD4 |
| **Naive T cell (CD4)** | 72.80 | 82.3-95.1 | % in CD4 |
| **Memory T cell (CD4)** | 27.20 | 4.6-17.3 | % in CD4 |
| **Active T cell (CD8)** | 2.90 | 0.4-4.4 | % in CD8 |
| **Naive T cell (CD8)** | 30.40 | 77.5-98.5 | % in CD8 |
| **Memory T cell (CD8)** | 69.60 | 1.0-11.1 | % in CD8 |
| **Relative B cell** | 31.10 | 16.3-26.8 | % in Ly |
| **Absolute B cell** | 1,534 | 871-1553 | per mm^3 |
| **Naive B cells** | 62 | 62-69 | % in B |
| **Immature peripheral B cell** | 20.10 | 9.7-17.9 | % in B |
| **IgM B cell** | 4 | 4.6-15.0 | % in B |
| **IgG B cell** | 1.1 | 1.5-4.2 | % in B |
| **IgA B cell** | 1.4 | 0.8-1.7 | % in B |
| **NK cell Relative** | 15 | 3-14 | % in Ly |
| **Absolute NK cell** | 717 | 100-1100 | per mm^3 |
| **Eosinophils** | 0.49 | 0.10-0.70 | x10^9/L |
| **Basophils** | 0.07 | 0-0.20 | x10^9/L |
| **Neutrophils** | 13.8 | 1.5-8.50 | x10^9/L |
| **Monocyte** | 2.05 | 0.00-0.80 | x10^9/L |
| **Lymphocyte** | 5.48 | 2-8 | x10^9/L |
| **Leukocytes** | 21.9 | 5.5-15.5 | x10^9/L |
| **IgM** | 1.3 | 0.1-0.87 | g/L |
| **IgA** | 1.2 | 0.19-1.1 | g/L |
| **IgG** | 12.7 | 2.6-13.9 | g/L |
| **IgE** | 176 | 0-32 | kU/L |

**Table S5. Normal response to routine 10-valent pneumococcal vaccination**

| **Polysaccharide antibodies*** | **Result** | **Unit** |
| --- | --- | --- |
| **anti-Pneumococcus type 6B** | N/A | µg/mL |
| **anti-Pneumococcus type 8** | 0.21 | µg/mL |
| **anti-Pneumococcus type 9V** | 1.9 | µg/mL |
| **anti-Pneumococcus type 14** | 2.3 | µg/mL |
| **anti-Pneumococcus type 15B** | <0.040 | µg/mL |
| **anti-Pneumococcus type 19F** | >40 | µg/mL |
| **anti-Pneumococcus type 20** | 0.13 | µg/mL |
| **anti-Pneumococcus type 23F** | 0.27 | µg/mL |
| **anti-Pneumococcus type 33F** | <0.040 | µg/mL |

*10 months after the third and final, routine vaccination with 10-valent pneumococcal vaccine (Synflorix), as part of the regular Dutch vaccination schedule.

**Table S6. Serology results of the index patient at age 3 years**

| **Virus** | **Result** | **Unit** |
| --- | --- | --- |
| Cytomegalovirus IgG | 17.1/Pos | ratio |
| **Diphtheria antibodies** | **0.55/Protective** | IU/mL |
| Epstein-Barr virus VCA IgG | 0.360/Neg | ratio |
| Epstein-Barr virus NA IgG | 0.363/Neg | ratio |
| **Hepatitis B virus anti-HBs** | **>1000.00/Protective** | IU/L |
| Influenza B virus antibodies | <1:10 | titer |
| Influenza A virus antibodies | <1:10 | titer |
| **Measles virus IgG** | **7366/Protective** | mIU/mL |
| **Mumps virus IgG** | **0.49/Neg** | ratio |
| **Poliovirus type 1 antibodies** | **1:128/Protective** | titer |
| **Poliovirus type 3 antibodies** | **1:384/Protective** | titer |
| **Rubella virus IgG** | **51.8/Protective** | IU/mL |
| SARS-CoV-2 | 0.04/Neg | ratio |
| **Tetanus toxin antibodies** | **1.34/Protective** | IU/mL |
| Varicella Zoster virus IgG | >2338/Pos | mIU/mL |

Pos = positive, Neg = negative

Vaccination induced responses are depicted in bold

**Supplementary figures**

**Figure S1. Gating strategy for granulocytes and monocytes in controls and index patient.**

Flow cytometry plot of whole blood of the index patient (top row) and an infant control patient (bottom row). Cells were gated based on their characteristic forward and side scatter properties, subsequently single and live cells (7-AAD^-^) were selected. Monocytes were identified as CD14^+^/CD16^-^; granulocytes were identified as CD14^-^/CD16^+^.

**Figure S2. Schematic view of CD14 mutation.**

a) Sanger sequencing of the CD14 gene revealed a single-nucleotide deletion in the CD14 gene (NM_000591.4:c.196del), for which the patient was homozygous. The wild type (top) and ΔC196 (bottom) alleles of the CD14 gene (left), and the proteins they encode (right) are schematically represented. The full-length CD14 protein consists of 375 amino acids (aa). The ΔC196 deletion is a frameshift mutation that changes the codon encoding leucine at position 66 into a stop codon (NP_000582.1:p.Leu66). (b) Characterization of the CD14 molecule with in green a prediction of the mutant form of CD14 in our patient, lacking the pocket rim for LPS binding.[4]

**Figure S3. Normal CD14 surface expression on immune cells of parents and sibling of the index patient.**

Flow cytometry plot of whole blood of both parents, sister and the index patient. Cells were gated based on their characteristic forward and side scatter properties, subsequently monocytes were identified based on CD33^+^/HLA-DR^+^ surface staining. On the bottom a histogram shows monocyte surface expression of CD14 in all family members, while this is absent in the patient.

**Figure S4: IL-6 and IL-8 levels in sputum of RSV patients**

Levels op IL-6 and IL-8 in sputum were measured by ELISA. Sputum was derived of the index patient and compared to control infants, samples were taken during severe RSV bronchiolitis (n=18).

**Figure S5.** **Impaired IL-6 production after prolonged stimulation of index patient PBMCs by TLR agonists LPS, but not R848.**

(a) IL-6 protein response to different concentration of LPS (E. coli) after 4, 18 and 24 hours of stimulation, by PBMCs of two healthy controls and the index patient. (b) IL-6 protein response to R848 1 µg/mL after 4, 18 and 24 hours of stimulation, by PBMCs of two healthy controls and the index patient.

**Figure S6. Confirmation of the CD14 knock out cell line.**

(a) Agarose gel images with standard (std; in base pairs) from wild-type (WT) and knockout (KO) CD14. (b) Primer sequences used for PCR and Sanger sequencing.

**Figure S7: IL-8 response by HNEC-ALI**

IL-8 production at day 0, 1, 2 and 3 post infection in basal medium after RSV or mock infection in WT and CD14 -/- HNEC- ALI.

**Figure S8.** **No LPS contamination of RSV-pre-F.**

(a) IL-6 production after 16 hours of stimulation with LPS (1 ng/mL) or RSV pre-F (5 µg/mL) in the presence or absence of polymyxin B (10 µg/mL). N = 3 experiments with a total of 5 donors. (b) Shows IL-6 production after 16 hours of stimulation with LPS (1 ng/mL) or RSV pre-F (10 µg/mL) after heat inactivation. N = 3 experiments with a total of 8 donors.

**Figure S9: Heatmap of gene expression by patient and healthy control PBMCs**

Gene expression by PBMCs after 4 hours of stimulation with a panel of TLR ligands, quantified using NanoString TechnologyTM. Log2 normalized counts are represented for the index patient (top), and a healthy control (bottom). Upregulated genes are depicted in red, downregulated genes in blue. Each row represents a stimulus. Each column represents one gene.

**Figure S10. Pulmonary computed tomography (CT) of index patient.**

CT-image of a cross-section of the lungs of the index patient at the age of 21 months. Red circles indicate areas suspected of ground-glass opacification in the left and right lung.

**Figure S11. Gating strategy for IgG and IgA positive memory B-cells.**

Gating strategy used to identify pre-F and post-F probe-binding IgG+ and IgA+ memory B-cells in human PBMCs. Lymphocytes were selected based on their forward and side scatter properties, live cells and surface expression of CD19^+^. Plasma blasts (CD38^++^CD27^++^) were excluded and populations were further gated on isotype-switched cells (IgM^-^IgD^-^) to identify probe-binding IgG^+^ and IgA^+^ memory B cells.

**Figure S12: epithelial cell differentiation of HNEC-ALI**

Relative gene expression of P63, FOXJ1 and MUC5AC by HNEC-ALI in WT (n=4) and CD14-/- (n=4) cultures measured by real-time quantitative PCR. Bars show mean +SD of two individual experiments with 2 donors per experiment.
